# Relationship Between Satisfaction and Concern with Own Appearance and Subjective Estimation of Economic Status

**DOI:** 10.3390/bs10010009

**Published:** 2019-12-19

**Authors:** Vera Labunskaya

**Affiliations:** Department of Social Psychology, Academy of Psychology and Pedagogy, Southern Federal University, M. Nagibina, 13, of 219, Rostov-on-Don 344038, Russia; vlab@aaanet.ru; Tel.: +7-918-555-5350

**Keywords:** appearance, concern and satisfaction with appearance, subjective evaluation of economic status

## Abstract

This study is aimed to scrutinize the relationship between satisfaction and concern with an individual’s physical appearance and the subjective estimation of economic status, which is considered as one of main predictors of life satisfaction. Recent research has focused on the role of real economic status on different aspects of human life, including satisfaction and concern with own appearance. In contrast with such studies, our investigation is focused on a subjective-comparative approach to economic status evaluation. According to this approach, the participants have to identify themselves either with the group “rather poor than rich” or with the group “rather rich than poor”. We hypothesize that satisfaction and concern with own appearance in youth varies as a factor of subjective evaluation of economic status. The participants were 41 male and 82 female undergraduate and graduate students of different majors, aged 20–35. Of the subjects, 62% estimated their economic status as “rather poor than rich” and 38% of participants identified themselves with the group “rather rich than poor”. The paper-pencil questionnaires “Attitudes towards Own Appearance: Satisfaction and Concern” and “Subjective Evaluation of Economic Status” were administered. Results indicates that subjective evaluation of economic status has a stronger association with satisfaction with one’s own physical appearance in comparison with concern over one’s own appearance.

## 1. Introduction

Recently, there has been a growth of investigations focusing on satisfaction and concern with one’s own physical appearance [1,2,3]. These studies analyze the impact of demographic factors as well as real and objective economic status on satisfaction and concern with appearance, body image, etc. [4,5,6,7,8,9]. Harris and Carr [10] revealed that the tendency to be concerned with and take care of appearance is higher in females in comparison with males, regardless of age. The authors also concluded that the intensity of concern with appearance does not depend on socio-economic status. At the same time, few studies showed that the association of socio-economic status with other demographic factors can influence the concern with appearance. Thus, Czyz et al. [7] found significant differences in body image of teenagers with low and high socio-economic status in South Africa: teenagers with high economic status reported higher discrepancies in body image in comparison with their counterparts of lower economic status. In addition, Chang et al. [6] showed that economic status is linked to experiences related to changes in appearance, body image, and life satisfaction. The studies demonstrated the association between such factors as employed/unemployed female, individual income, family income, married/unmarried female, education, on the one hand, and experiences related to changes in appearance, body image, and life satisfaction, on the other. Summarizing these findings, employed females with an independent source of income reported a higher concern with appearance and “poorer” body image compared with unemployed females. Further, higher family income correlated significantly with positive body image. In the study by Benson et al. [5], economic status is linked to self-respect and evaluation of life quality depending on the quality of teeth. In another study, such factors as social anxiety and loneliness were included in the analysis of the relationship between socio-economic status and appearance [4]. If social anxiety and loneliness were included as additional factors, the results indicated a nonsignificant influence of social status with regard to concern with appearance. In addition, Dea and Caputi [9] studied the interplay between socio-economic status and such factors as gender, age, weight, body image, and practices of weight control. The findings revealed that children, teenagers, and young people with lower economic status showed a higher tendency to be overweight. It was also demonstrated that lower socio-economic status does not impact the evaluation of physical appearance in spite of the tendency of this participant group to be overweight. In a study by Francis [8] evaluating the association between economic status and perception of clothing deprivation in high school students, lower clothing deprivation was more characteristic for students with lower economic status.

Summing up, socio-economic status has a significant impact on attitudes towards one’s own appearance. Results of studies mentioned above demonstrate the importance of analyzing the interplay of socio-economic status with other factors, involving perception and evaluation of appearance. One limitation of the above-named studies relates to different methods of measuring socio-economic status. In this regard, the psychological and, especially, social-psychological research paradigm suggests the inclusion of the perspective of the single individual and to stress the factors that reflect specific individual activities directed at changes in appearance and, consequently, at changes in satisfaction and concern with appearance [2]. Thus, combining individual factors with other relevant factors related to satisfaction and concern with appearance can provide a promising approach in analyzing the interrelations between physical appearance and life perspective in youth in Russia.

According to Khashchenko et al. [11,12], a large part of studies focusing on the attitudes to economic inequality in Russia were conducted at the beginning of the twenty-first century as social groups were formed and defined in relation to their economic characteristics that, in turn, offered a basis for self-categorization. The most attention was paid to subjective evaluation of own economic status and identification with categories “poor” and “rich”. Khashchenko et al. [11,12] defined subjective economic well-being as a system of attitudes towards different aspects of personal economic situations. This system of attitudes varies in relation to personal values, goals, well-being standards, and self-estimation from the perspective of an economic individual. Subjective evaluation of economic status contains evaluation of financial status, economic identity, as well as comparative categorization based on the criteria “poor/rich” [13]. 

On the basis of the approach describe above with regard to economic status evaluation, we developed a new subjective-comparative approach to studying economic status evaluation that reflects individual’s self-identification either with the group “rather poor than rich” or with the group “rather rich than poor”. The definition of appearance and satisfaction/concern with appearance is presented in additional detail in our previous research [1,2,3]. According to this definition, satisfaction and concern with appearance is considered independent phenomena. Therefore, the present research is aimed at analyzing the association between subjective evaluation of economic status that is operationalized as self-identification with “rather poor than rich” group or “rather rich than poor” group and satisfaction and concern with appearance as well as with a wish to change one’s appearance. The rest of the paper is organized as follows: the next section presents the materials and the methods, Section 3 presents the results, and the last section concludes the findings and discusses the limitations of this research.

## 2. Materials and Methods

It is hypothesized that (1) satisfaction and concern with appearance in youth vary as a function of subjective evaluation of economic status, and (2) there is a relationship between gender and subjective evaluation of economic status, on the one hand, and satisfaction and concern with appearance as well as wish to change the appearance, on the other hand. The following steps will be realized to test the hypotheses: (1) the groups of participants with the self-identifications “rather poor than rich” and “rather rich than poor” will be defined; (2) the level of satisfaction and concern with the participant’s own appearance as well as wish to change their appearance will be measured; (3) the relationship between subjective evaluation of economic status and satisfaction and concern with appearance and wish to change the appearance will be estimated; (4) the association between gender and satisfaction and concern with appearance and wish to change one’s appearance will be assessed; (5) the impact of interplay between subjective evaluation of economic status and gender on satisfaction and concern with appearance and wish to change one’s appearance will be analyzed. The data was processed using SPSS 24. T-tests for one sample and for independent samples were applied to estimate the differences in levels of satisfaction and concern with appearance as well as the wish to change the appearance as dependent on gender and self-reported economic status. MANOVA was used to calculate the interaction effect of economic status and gender on appearance attitudes.

The participants included 41 male and 82 female students at undergraduate (35%) and postgraduate (65%) levels aged 20–35 (M = 27.6, SD = 4.2) with majors in Psychology (50%), Educational Sciences (25%), and Philology (25%). Participants were recruited on a volunteer basis and received no incentives for their participation in the study. Two paper-pencil questionnaires “Subjective Evaluation of Economic Status” and “Attitudes towards Own Appearance: Satisfaction and Concern” [1] were administered (information concerning validation of the questionnaires could be requested from the author). The questionnaire “Subjective Evaluation of Economic Status” contains two dimensions “rather poor than rich” or “rather rich than poor”. When filling out the questionnaire, participants are required to indicate the economic status they identify themselves with. According to the frequency scores, the participants’ subjective evaluation of economic status can be coded as “rather poor than rich” or “rather rich than poor”. Further, the questionnaire “Attitudes towards own appearance: satisfaction and concern” [1] includes 12 items that are organized according to three scales. Concern with appearance was represented through items discussing feelings of discomfort, unease, and embarrassment experienced in relation to own appearance in different interaction situations. Satisfaction with appearance was represented through the items associated with the impression one’s appearance has on other people. The tendency towards appearance change was operationalized with such items as “How often you invest time in appearance change with the aim to positive impress others?” Participants were asked to indicate the degree of their agreement with statements on a 10-point Likert scale. For each scale, the average intensity score was calculated. The internal consistency of the questionnaire was satisfying, with Cronbach’s alpha varying between =.54 and =.92 for three scales. The study was ethically approved through the ethical review committee of the Academy of Psychology and Pedagogy of the Southern Federal University.

## 3. Results

### 3.1. Participants with Self-Identifications “Rather Poor than Rich” and “Rather Rich than Poor”

Out of the subjects tested, 62% estimated their economic status as “rather poor than rich” and 38% of participants identified themselves with the group “rather rich than poor”. In both economic status groups, male and female participants were represented equally. Of the male participants, 30% and 32% of the female participants identified with the group “rather poor than rich”. In comparison, 20% of male participants and 18% of female participants reported the identification with the economic status “rather rich than poor”. The results show that higher amount of participants perceive their economic status as “rather poor than rich”, which reflects the financial situation of youth.

### 3.2. Intensity of Attitudes towards Onw Appearance

The analysis of the relationship among the three studied attitudes towards own appearance revealed significant negative correlation between satisfaction with appearance and concern with appearance (r (122) = −0.32, *p* < 0.01) and positive significant correlations between both satisfaction and concern with appearance and wish to change the appearance (r (122) = 0.39, *p* < 0.01 and r (122) = 0.18, *p* = 0.04, respectively). As the three appearance scales are normally distributed as assessed by the Shapiro-Wilk-Test (all p- values are smaller than 0.05: *p* = 0.06 for satisfaction with appearance, *p* = 0.15 for concern with appearance, *p* = 0.07 for wish to change one’s appearance), the T-test for one sample was applied to analyze the differences in the intensity level of three attitudes towards own appearance. The mean of each of the three scales was used as the test value against which the intensity of each attitude was compared in a paired manner. The results indicated a significant difference in the intensity of all three attitudes towards own appearance with satisfaction with appearance (M = 7.07, SD = 1.45) over a wish to change one’s appearance (M = 6.03, SD = 1.98; t (121) = 5.91, *p* < 0.001) and concern with appearance (M = 5.19, SD = 1.75; t (121) = 15.02, *p* < 0.001) as well as significant differences between wish to change the appearance and concern with appearance (t (121) = −6.95, *p* < 0.001), as seen in Table 1. The analysis of scores revealed that satisfaction with appearance is significantly higher than concern with appearance and wish to change one’s appearance. At the same time, a wish to change one’s appearance is significantly higher than concern with appearance. Taken together, for young people in Russia, it is characteristic to be highly satisfied with appearance and to show a high tendency to change their appearance. 

### 3.3. Self-Reported Economic Status and Attitudes towards Onw Appearance

The T-test for independent variables was used to compare the difference in the intensity of appearance attitudes dependent on gender and self-reported economic status. The analysis of the relationship between subjective evaluation of economic status and attitudes towards own appearance demonstrated that participants with self-identification “rather rich than poor” reported significantly higher satisfaction with their appearance (M = 7.52, SD = 1.28; t (120) = −2.68; *p* = 0.008) and a higher wish to change own appearance (M = 6.9, SD = 1.82; t (120) = −2.48; *p* = 0.014) in comparison to participants with self-identification “rather poor than rich” (M = 6.8, SD = 1.49; M = 6.0, SD = 2.01), as seen in Table 2. The intensity of concern with appearance in both participants groups did not differ significantly.

Summing up, the satisfaction with appearance and the wish to change one’s appearance vary significantly as a function of subjective evaluation of economic status but not with regard to a concern with one’s appearance. 

### 3.4. Gender and Attitudes towards Onw Appearance

The analysis of the differences in intensity of appearance attitudes in relation to gender allows to conclude that female participants report significantly higher concern with appearance (M =5.43, SD = 1.77) in contrast with male participants (M = 4.72, SD = 1.64; t (120) = −2.11; *p* = 0.036). There were no significant differences in the intensity of satisfaction with appearance and wish to change own appearance in female and male participants (Table 3).

On the basis of the reported results, it could be concluded that both female and male participants are satisfied with their appearance and show the tendency to change their appearance in a similar way. Thus, concern with appearance predominantly varies as a factor of gender.

### 3.5. Impact of Self-Reported Economic Status and Gender on Attitudes towards Onw Appearance

**Figure 1:**
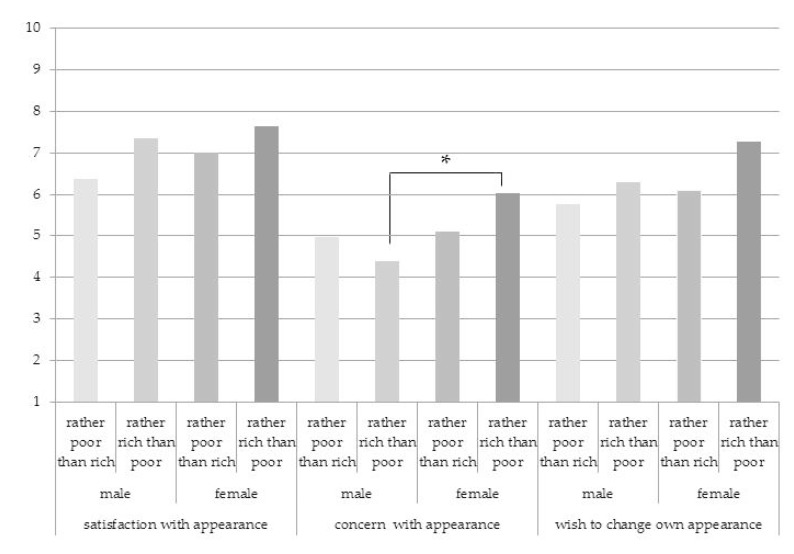
Impact of interplay between gender and subjective evaluation of economic status on satisfaction with appearance, concern with appearance, and wish to change the appearance. * significant differences at the *p*-value level of ≤ 0.05 are indicated with asterisk.

The analysis of the impact of interplay between gender and subjective evaluation of economic status, on the one hand, and attitudes towards appearance, on the other hand, showed that there is only one significant interaction effect of gender and self-assessed economic status on concern with appearance. In contrast with male participants, female participants with the self-identification “rather rich than poor” report higher intensity on concern with appearance (*F* (1, 122) = 5.06, *p* = 0.026), as seen in Figure 1. However, the interaction effect of gender and self-assessed economic status on satisfaction with appearance and wish to change own appearance was not significant (*F* (1, 122) = 0.37, *p* = 0.54 and *F* (1, 122) = 0.68, *p* = 0.40, respectively).

## 4. Discussion and Conclusions

We could provide more detailed analysis of the individual’s perception of socio-economic status due to implementation of subjective-comparative approach. Further, we scrutinize the role of subjective evaluation of economic status with regard to intensity of satisfaction and concern with appearance as well as a wish to change one’s appearance. Research participants with self-identification “rather rich than poor” report higher satisfaction with appearance and a higher wish to change their appearance in comparison with research participants with the self-identification “rather poor than rich”. Subjective evaluation of economic status does not significantly influence concern with appearance in the two groups of participants. 

The distribution between female and male participants in the group “rather poor than rich” does not differ significantly from the group “rather rich than poor”. Both male and female participants, independent from self-evaluated economic status, show similar satisfaction with appearance and wish to change their appearance. Additionally, we revealed that males in the group “rather rich than poor” demonstrate significantly lower concern with appearance when compared with females in this economic status group. 

In conclusion, the factor of gender has to be included in the analysis of the impact of subjective evaluations of economic status on satisfaction and concern with appearance in youth. The findings testify the formulated hypotheses and indicate that the relationship between gender factors and subjective evaluation of economic status are significantly associated with satisfaction and concern with appearance as well as with a wish to change one’s appearance. Future studies could also analyze more deeply the interplay of other demographic parameters, such as age, with socio-economic status and gender on appearance attitudes.

The results could be also interpreted from the perspective of health issues and, especially, dental health as a part of physical appearance. As mentioned in some recent studies [14,15], healthy physical appearance, such as oral health, is connected to psychological well-being and therefore impacts quality of life in individuals and their participation in societal life. 

The findings of this research contribute to a more differentiated analysis of the impact of social-psychological factors on satisfaction and concern with appearance [4,5,6,7,8,9]. The direct association between socio-economic status and concern with appearance could not be confirmed fully in our research [10]. At the same time, the results of our research testify to the findings of numerous studies demonstrating the impact of gender on satisfaction and concern with appearance.

## Figures and Tables

**Table 1:** Differences in intensity of satisfaction with appearance, concern with appearance, and wish to change one’s appearance.

Scales	t	Sig.(2-Tailed)
satisfaction with appearance–concern with appearance	15.022	0.000
satisfaction with appearance–wish to change the appearance	−6.95	0.000
Concern with appearance–wish to change the appearance	5.91	0.000

**Table 2:** Satisfaction with appearance, concern with appearance, and wish to change own appearance in relation to subjective evaluation of economic status.

Scales	Self-Evaluated Socio-Economic Status	N	Mean	Standard Deviation	t	Sig. (2-Tailed)
satisfaction with appearance	rather poor then rich	76	6.81	1.49	−2.68	0.008
	rather rich then poor	46	7.52	1.28		
concern with appearance	rather poor then rich	76	5.05	1.78	−1.10	0.27
	rather rich then poor	46	5.42	1.69		
wish to change own appearance	rather poor then rich	76	6.00	2.01	−2.48	0.014
	rather rich then poor	46	6.90	1.82		

**Table 3:** Satisfaction with appearance, concern with appearance, and wish to change own appearance in relation to participants’ gender.

Scales	Gender	N	Mean	Standard Deviation	t	Sig. (2-Tailed)
satisfaction with appearance	male	41	6.77	1.56	−1.648	0.102
	female	81	7.23	1.38		
concern with appearance	male	41	4.72	1.64	−2.118	0.036
	female	81	5.43	1.77		
wish to change own appearance	male	41	5.98	2.03	−1.399	0.164
	female	81	6.51	1.94		
